# Exploring patient willingness to accept hepatitis C-infected kidneys for transplantation

**DOI:** 10.1186/s12882-020-02114-y

**Published:** 2020-11-10

**Authors:** Gretchen C. Edwards, Maren E. Shipe, Lindsay Smith, Christianna Gamble, David Shaffer, Beatrice P. Concepcion, Rachel Forbes

**Affiliations:** 1 Department of General Surgery, Vanderbilt University Medical Center, Medical Center North, Suite CCC-4312, 1161 21st Avenue South, Nashville, TN 37232-2730 USA; 2 Vanderbilt University Medical Center Transplant Center, Nashville, USA; 3 Department of General Surgery, Division of Kidney and Pancreas Transplantation, Vanderbilt University Medical Center, Nashville, USA; 4 Department of Medicine, Division of Nephrology and Hypertension, Vanderbilt University Medical Center, Nashville, USA

**Keywords:** Hepatitis C, PHS-high risk, Expanded donor criteria, Deceased donor kidney transplant, Disparities

## Abstract

**Background:**

As organs infected with Hepatitis C virus (HCV) provide an opportunity to expand the donor pool, the primary aim of this study is to explore patient willingness to accept a kidney from HCV-infected donors compared to other high-risk donors.

**Methods:**

An anonymous, electronic survey was sent to all active kidney transplant waitlist patients at a single large volume transplant center. Patients were asked to respond to three hypothetical organ offers from the following: 1) HCV-infected donor 2) Donor with active intravenous drug use and 3) Donor with longstanding diabetes and hypertension.

**Results:**

The survey was sent to 435 patients of which 125 responded (29% response rate). While 86 out of 125 patients (69%) were willing to accept an HCV-infected kidney, only a minority of respondents were willing to accept a kidney from other high-risk donors. In contrast to other studies, by multivariable logistic regression, age and race were not associated with willingness to accept an HCV-infected kidney.

**Conclusions:**

In this exploratory study, utilization of kidneys from HCV-infected donors to expand the donor pool appears to be an acceptable option to patients.

**Supplementary Information:**

The online version contains supplementary material available at 10.1186/s12882-020-02114-y.

## Background

There is a critical shortage of donor organs for patients awaiting kidney transplantation. According to the Organ Procurement and Transplantation Network (OPTN), nearly 100,000 patients remain on the kidney transplant waiting list in the United States (U.S.) and yet only 10,000 patients underwent deceased donor kidney transplantation in 2018 [1]. As the demand for organs continues to outpace supply and waiting times exceed up to 5 years in many parts of the country, multiple approaches are now being considered to safely expand the donor pool [2]. These include donation after circulatory death, public health service (PHS) increased risk donors, older donors with comorbidities, and those previously exposed to or infected with hepatitis C (HCV).

The ongoing opioid epidemic in the U.S. has resulted in a dramatic increase in the number of deaths due to overdose with intravenous drug use (IVDU), leading to a significant increase in donors classified as PHS increased risk and those infected with HCV [3]. With the advent of direct-acting antiviral agents (DAA) for the treatment of HCV, there has been great interest in the potential to increase the donor pool by offering waitlisted patients HCV-infected kidneys. The cure rates of various DAA regimens exceed 90% in the general population and evidence is growing to suggest that similarly high cure rates can be achieved among immunosuppressed recipients and those with renal dysfunction [4–8]. Notably, two independent single-center pilot studies demonstrated 100% cure rates and excellent outcomes in a total of 30 uninfected patients who received HCV-infected kidneys and were subsequently treated with DAA post-transplant [9, 10]. More recently, others have reported excellent outcomes on this practice when performed as standard of care, i.e. outside of a clinical trial [11, 12].

Despite the growing interest in the transplant community to broadly adopt the practice of transplanting HCV-infected kidneys into uninfected recipients, little is known about patient willingness to accept such kidneys and how this compares to willingness to accept other “non-standard” kidneys such as PHS increased risk and high kidney donor profile index (KDPI).

The aims of this study were: First, to quantify patient willingness to receive a kidney from a donor with HCV-viremia; second, to compare this with willingness to accept a kidney from a representative PHS increased risk or marginal (high KDPI) donor; and third, to identify clinical characteristics associated with willingness to accept an HCV-infected kidney.

## Methods

### Participants and survey administration

An anonymous, electronic survey was administered to all active kidney transplant waitlist patients with a registered email address at a large volume transplant center in Nashville, TN as part of an exploratory initiative to gauge interest among waitlisted patients in accepting HCV-infected kidneys. As such, no pilot survey was conducted at the initiation of the study. However, all study materials were reviewed by transplant center staff who regularly produce educational and survey materials for this study population. Multiple iterations were circulated among transplant center including surgery, nephrology, nursing, and administrative staff. Furthermore, the survey was set up in a similar fashion to the hypothetical situations posed by McCauley, et al., whose work was based on semi-structured interviews [13]. Survey data were collected via the REDCap electronic data capture tool. Patients were provided introductory information to the survey, including observed HCV cure rates with current DAA therapy, and were informed that participation was completely voluntary. All patients had previously undergone in-person pre-transplant education at the transplant center which included education regarding the risks and benefits of PHS-increased risk and high KDPI kidneys, but not HCV-infected kidneys. This study was deemed exempt under the Institutional Review Board (IRB # 190290).

### Demographic and clinical data

Patients were asked for demographic information (age, sex, race, ethnicity, education level), waitlist time, dialysis time, type of dialysis, and history of a prior kidney transplant. Patients were also asked if they had been diagnosed and/or treated for HCV and if they had any personal contacts with known history of HCV. They were then asked to respond to three hypothetical kidney offers as follows: 1) 20-year-old HCV-infected donor with a greater than 95% chance of successful HCV cure following transplant; 2) 20-year-old PHS-increased risk donor with active IVDU at time of death; and 3) 70-year-old donor with long-standing hypertension and diabetes. For those patients who reported “no” to any organ offer, they were then asked to indicate how much additional time they would be willing to remain on the transplant list in order to receive a “standard” kidney offer. Finally, patients were asked if added costs or doctor visits for HCV treatment were of concern to them. A full copy of the survey is included as Additional file 1.

### Statistical analysis

This study was designed as an exploratory survey to gauge attitudes regarding willingness to accept HCV-positive organs and to shape further educational and outreach efforts regarding these organ offers. Thus, no sample size calculation was performed. Descriptive analysis including mean and median for continuous variables and frequencies for categorical variables were calculated. Student *t* test and one-way analysis of variance (continuous variables) and Fisher’s exact test (categorical data) were used to compare differences between groups. Multivariable logistic regression was utilized to identify clinical characteristics which were independently associated with willingness to accept an HCV-infected kidney. Covariates included in the model were chosen a priori and included age, race, gender, educational status, and time on dialysis. Time on dialysis was categorized to “less than 2 years” and “greater than or equal to 2 years” while educational status was categorized as “some college or less” and “graduated college, graduate or doctoral degree”. All analyses were performed using Stata 15.1 (StataCorp, College Station, TX).

## Results

A total of 435 patients who were actively listed for kidney transplant at our center and had a registered email address were sent the electronic link to the REDCap survey. There were 125 patients who responded (29% response rate). The demographic and clinical characteristics of the cohort are presented in Table 1. The mean age was 55 years, 57% were male, 66% were white, and 28% were black. Of the patients who responded, 70% are currently on dialysis, the majority of whom undergo hemodialysis (66%). Time on dialysis as well as time on transplant list (years) are also shown in Table 1.

**Table 1:** Demographic and clinical characteristics of survey participants (*n* = 125)

	Overall	Unwilling to receive HCV+ kidney (***N*** = 39)	Willing to receive HCV+ kidney (***N*** = 86)	***P***-value
Mean age (years) +/− SD	55 +/− 1.06	51 +/− 2.3	56.7 +/− 1.1	0.012
Male sex, N (%)	71 (56.8)	20 (51.3)	51 (59.3)	0.402
Race				
White, N (%)	82 (65.6)	25 (64.1)	57 (66.3)	
Black, N (%)	35 (28.0)	11 (28.2)	24 (27.9)	
Other, N (%)	8 (6.4)	3 (7.7)	5 (5.8)	0.919
Currently on dialysis, N (%)	88 (70.4)	30 (77.0)	58 (67.4)	0.282
Two or more years on dialysis, N (%)	44 (35.2)	11 (28.2)	33 (22.1)	0.072
Dialysis type, N (%)				
Hemodialysis	58 (65.9)	19 (48.7)	39 (45.3)	
Peritoneal dialysis	30 (34.1)	11 (28.2)	19 (22.1)	0.174
Mean number of years listed, +/− SD	1.9 +/− 0.15	1.9 +/− 0.27	2.0 +/− 0.18	0.66
History of prior kidney transplant, N (%)	19 (15.2)	8 (20.5)	11 (12.8)	0.265
Personal history of HCV, N (%)	6 (4.8)	1 (2.6)	5 (5.8)	0.424
Previously treated for HCV, N (%)	6 (4.8)	1 (2.6)	5 (5.8)	0.424
Know someone with HCV, N (%)	20 (16.0)	7 (17.9)	13 (15.1)	0.689
Educational level, N (%)				
Some high school	4 (3.2)	0 (0)	4 (4.7)	
High school diploma or GED	19 (15.2)	4 (10.3)	15 (17.4)	
Some college	43 (34.4)	13 (33.3)	30 (34.9)	
Graduated college	37 (29.6)	10 (25.6)	27 (31.4)	
Graduate or doctoral degree	22 (17.6)	12 (30.8)	10 (11.6)	0.071
Interested in hearing more about HCV-positive organs	75 (60.0)	5 (12.8)	70 (81.4)	< 0.001

**Fig. 1:**
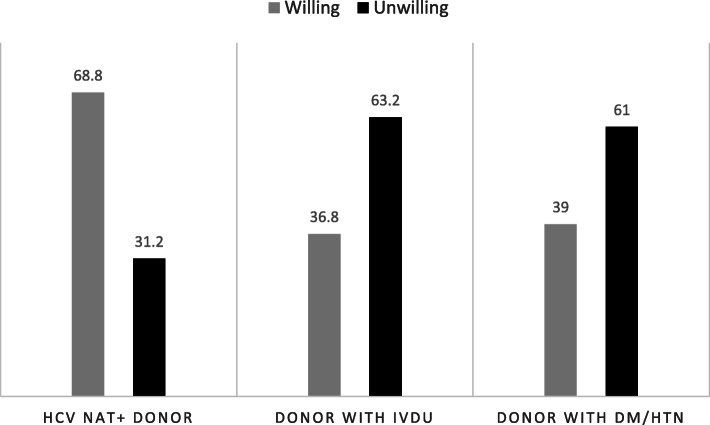
Willingness (%) to accept three hypothetical organ offers: 1) 20-year old HCV NAT+ donor, 2) 20-year-old donor with IVDU at time of death, or 3) 70-year old donor with long-standing diabetes and hypertension

Out of 125 respondents, 86 (69%) were willing to accept an HCV-infected kidney. In unadjusted analysis, those patients willing to receive HCV-infected kidneys were significantly older (57 versus 51 years old, *p* = 0.012). There was no significant difference in willingness to accept HCV-infected kidneys by sex, race, time on dialysis, or educational status (Table 1). In contrast to patient willingness to accept an HCV-infected kidney, only a minority of respondents were willing to accept a kidney from a donor with active IVDU at the time of death (37%) or from an older donor with long-standing history of diabetes and hypertension (39%) (Fig. 1).

In multivariable logistic regression analysis, neither age (OR 1.03, 95% CI 0.99–1.07) nor race (OR 0.63, 95% CI 0.24–1.68) were significantly associated with willingness to accept HCV-infected kidneys after adjustment for sex, time on dialysis, or educational status. (Table 2).

**Table 2:** Multivariable logistic regression model for willingness to accept an HCV-infected kidney

Question: Willing to receive organ from HCV NAT+ donor	Odds Ratio	95% CI	***P***-value
Age	1.03	0.99–1.07	0.13
Sex (Ref: Male)	0.84	0.31–2.27	0.74
Race (Ref: White)	0.63	0.24–1.68	0.36
≥ 2 years on dialysis (Ref: < 2 years on dialysis)	2.08	0.79–5.52	0.14
Educational status college or graduate degree (Ref: Some college or less)	0.42	0.16–1.11	0.08

For those patients who reported “no” to any organ offer and were subsequently asked to respond to how much additional time they would be willing to wait in order to receive a “standard” kidney offer, those who declined an HCV-infected kidney were willing to wait for a mean additional time of 3.5 years. Finally, 38% of patients were concerned about the additional costs of HCV medications as well as the burden of additional office visits for HCV treatment.

## Discussion

In an exploratory survey conducted in a large volume transplant center in the Southeastern United States, we found that most patients were willing to accept an offer for an HCV-infected kidney. In contrast to prior studies, our data suggest that there were no significant differences in willingness to accept offers for HCV-infected kidneys based on age and race. Our finding that majority of patients are interested in accepting an HCV-infected kidney is similar to what other groups who have found in their own patient population. However, in contrast to our findings, these studies found that older patients and white patients were more willing to accept HCV-infected kidneys than younger and black patients, respectively [13, 14]. The reason for the contrast in the results is unclear but may represent unmeasured differences in each transplant center’s characteristics and patient population.

While our study demonstrates that many patients are open to HCV-infected kidneys once educated regarding the safety and efficacy of DAA therapy post-transplant, they also suggest that other types of “high-risk” donation is less clear to patients. The majority of our respondents were unwilling to receive an organ from a donor with active IVDU at the time of death, albeit with negative HIV and HCV serologies. Similarly, the majority of our respondents were unwilling to receive an organ from an older donor with diabetes and hypertension, two disease states contributing to end-stage renal disease. In these scenarios, there may be an element of uncertainty regarding expected outcome for both the patient and the provider.

This lack of certainty is clearly problematic, as the designation of PHS-increased risk leads to non-utilization of hundreds of organs every year [15]. In focus groups, Ros, et al. found that patients desired additional information about PHS-increased risk donors, including behaviors, kidney quality, and probability of undetected infection. This study also found that patients heavily weighed the opinion of their transplant provider in making recommendations regarding organ offers [16].

Participants in our survey received a modest amount of educational material regarding HCV-infected kidney offers and treatment prior to completing this anonymous survey. Given these data and other groups’ findings that patients prominently consider the opinion of the transplant provider in whether or not to accept an organ offer, we suggest that providers carefully consider the risks and benefits of an organ offer with their patients. These discussions should include consideration of patient age, current quality of life, and ability to detect and treat potentially transmitted infections.

This study has several limitations. First, our response rate was low (29%), participation was voluntary, and the sample size was limited. In order to more fully validate our results, a larger study cohort will be necessary. Therefore, it is possible that our findings are influenced by non-response bias and that patients who are interested in HCV-infected kidneys would be more likely to respond to this survey. Second, all respondents are listed at a single academic institution, which may limit the generalizability of the study. Third, the survey was designed to primarily gauge interest in accepting HCV-infected kidneys, hence no additional education within the survey was provided regarding the risks and benefits of accepting a PHS-increased risk or high KDPI kidney. While patients did receive in-person education regarding these kidneys during their evaluation at our transplant center, an important future direction of the study includes providing more focused educational material to respondents regarding each type of organ offer. Additionally, a full qualitative evaluation of the study questions within a pilot group before expanding to a larger cohort will be an important additional step in validating these findings. Finally, due to the limited number of events and to avoid overfitting the model, some other potential confounders may not have been included in the multivariable analysis.

## Conclusions

This exploratory study suggests that patients currently on the waitlist for kidney transplant are willing to consider organ offers from HCV-infected kidneys, but may be less accepting of organ offers from other “high-risk” donors without additional education and information about such offers. While the results of this initial survey are limited, these results suggest a need for each transplant center to survey their own transplant population. In order to validate the results of this preliminary work, qualitative validation of the study questions and a larger study cohort will be necessary. Equipping patients and providers alike with more tools to navigate decision-making regarding higher-risk donors remains an area of ongoing research.

## Supplementary Information

**Additional file 1.**

## Data Availability

The datasets used and/or analyzed during the current study are available from the corresponding author on reasonable request.

## Abbreviations

HCV: Hepatitis C Virus
PHS: Public Health Service
OPTN: Organ Procurement and Transplantation Network
IVDU: Intravenous drug use
DAA: Direct-acting antiviral agents
KDPI: Kidney donor profile index
