# Supplementary material for: Exploring patient willingness to accept hepatitis C-infected kidneys for transplantation
Source: BMC Nephrol. 2020 Nov 10;21:473. doi: 10.1186/s12882-020-02114-y (PMC7653701; doi:10.1186/s12882-020-02114-y)
Supplement: Supplementary file 1 — Additional file 1. [file 12882_2020_2114_MOESM1_ESM.docx]

**APPENDIX A**

*Introduction*

The Kidney Transplant Center at Vanderbilt University Medical Center is now expanding its program to offer kidneys from donors infected with Hepatitis C. Despite good treatment for Hepatitis C, we estimate that over 500 kidneys from these donors are discarded in the United States every year.

We are sending out an email survey to ask the patients currently on the kidney transplant list about their thoughts regarding kidneys from donors with Hepatitis C. This survey is entirely voluntary and will not impact your status on the waiting list. If you are interested in hearing more about this option, you will have the opportunity on the survey to indicate that you would like to speak to one of our transplant doctors about this.

Here is some general information about hepatitis C and kidney transplants that may be educational to you before you complete the survey:

Hepatitis C and Kidney Transplants:

- Kidney transplants improve daily health for people with severe kidney disease and help them to live longer.
- However, there are not enough kidneys for all the people who need them. Most patients with severe kidney disease spend years on the waiting list before they receive a kidney.
- There are risks to spending a long time on the transplant waiting list, such as feeling tired, becoming too sick be transplanted, or even dying.
- One way to get a kidney transplant sooner is to accept a kidney from someone who is infected with a virus, like hepatitis C.

What is hepatitis C?

- Hepatitis C is a virus that infects the liver. It is spread through blood. Hepatitis C infection can be short term, but usually hepatitis C is a chronic (long-term) disease.
- Most people who live with hepatitis C infection do not feel sick for a long time; often, they don’t even know they have the infection.
- Unfortunately, if left untreated for many years, hepatitis C often leads to liver failure, which can be deadly and require a liver transplant. Hepatitis C can also cause liver cancer.
- With new medications, doctors can often cure hepatitis C. We now have medications which can cure people of hepatitis C more than 95% of the time.

What is the impact of getting a kidney from someone who is infected with hepatitis C?

- It’s very likely that all people who received a kidney with hepatitis C would also be infected with hepatitis C after the transplant. Patients would be treated right away after the transplant. The treatment would likely cure most patients within 3 months.
- The Vanderbilt heart and liver transplant programs have transplanted approximately 100 HCV negative patients with HCV positive organs, with great success. The VUMC heart transplant cohort is the largest of its kind in published literature.

What do we want to know?

- Of course, everyone would prefer to receive a kidney from a completely healthy donor, but what if that meant you couldn’t get a kidney at all?
- We are interested in learning more about whether you would be willing to accept a kidney that was donated by someone who had hepatitis C or if you prefer to wait for a kidney donated by someone who did not have hepatitis C.

------------------------------------------------------

*General Demographic Information*

1. Age
2. Sex

- Male
- Female
- Prefer not to specify

1. Race

- White
- Black
- Asian
- Other
- Prefer not to specify

1. Ethnicity

- Hispanic/Latino
- Non-Hispanic/Latino
- Other
- Prefer not to specify

1. Are you on dialysis? (Y/N)
2. If yes, how many years have you been on dialysis?

- < 1 year
- 1-2 years
- 2-5 years
- > 5 years

1. If yes, what type of dialysis?

- Peritoneal dialysis
- Hemodialysis

1. How many years have you been on the kidney transplant list?
2. Have you had a kidney transplant in the past? (Y/N)
3. If yes, in what year did you have your kidney transplant?
4. Have you ever been told you have hepatitis C? (Y/N)
5. If yes, have you been treated for your hepatitis C? (Y/N)
6. Do you know anyone who has hepatitis C? (Y/N)
7. What is the highest level of education you have reached?
   - Less than high school
   - Some high school
   - High school graduate
   - Some college
   - College graduate
   - Graduate degree
8. Are you interested in hearing more about this program? (Y/N)
9. If yes, what kind of information would be most helpful and convenient for you to learn more about kidney transplants from hepatitis C donors? Please mark all that apply.
   - Information packet by email
   - Information packet by mail
   - Information session with transplant center staff
   - Phone conversation with transplant doctor
   - In-person appointment with transplant doctor
   - *Other (indicate here): ____________________*

*---------------------------------------------*

*Questions*

1. Your doctor calls to offer you a kidney transplant from a 20-year-old donor with hepatitis C infection. The doctor says there is > 95% chance that you will be successfully treated for hepatitis C after your transplant. This means that you would likely be cured of the hepatitis C contracted from the kidney transplant.
   1. Would you accept this kidney? (Y/N)
   2. If not, how many years would you be willing to wait in order to receive a kidney from a donor without hepatitis C?
      - 1 year
      - 2 years
      - 3 years
      - 4 years
      - > 5 years
2. Doctors call some organ donors “high risk” because they engaged in activities in the past which put them at a higher risk of having infectious diseases like HIV or hepatitis. All donors are screened for these diseases but some of our tests cannot detect the diseases if the patient was infected in the last two weeks.

Your doctor calls to offer you a kidney transplant from a 20-year-old donor who does not test positive for HIV or hepatitis. However, this donor was found to be injecting IV drugs at the time of their death. Therefore, your doctor cannot guarantee that they did not recently become infected with HIV or hepatitis.

- 1. Would you accept this kidney?
  2. If not, how many years would you be willing to wait in order to receive a kidney from a donor who was not using drugs at the time of their death?
     - 1 year
     - 2 years
     - 3 years
     - 4 years
     - > 5 years

1. Your doctor calls you to offer you a kidney transplant from a 70-year old donor who does not have hepatitis C, HIV, and was not engaged in high risk activities. This donor had high blood pressure for 20 years and diabetes for 10 years. Both high blood pressure and diabetes are known to cause damage to the kidneys.
   1. Would you accept this kidney?
   2. If not, how many years would you be willing to wait in order to receive a kidney from a donor who did not have high blood pressure and diabetes?
      - 1 year
      - 2 years
      - 3 years
      - 4 years
      - > 5 years
